# Spin-bearing molecules as optically addressable platforms for quantum technologies

**DOI:** 10.1515/nanoph-2024-0420

**Published:** 2024-10-24

**Authors:** Senthil Kumar Kuppusamy, David Hunger, Mario Ruben, Philippe Goldner, Diana Serrano

**Affiliations:** Institute for Quantum Materials and Technologies (IQMT), Karlsruhe Institute of Technology (KIT), Karlsruhe, Germany; Physikalisches Institut, Karlsruhe Institute of Technology (KIT), Karlsruhe, Germany; Institute of Nanotechnology, Karlsruhe Institute of Technology (KIT), Karlsruhe, Germany; Centre Européen de Sciences Quantiques (CESQ), Institut de Science et d’Ingénierie Supramoléculaire (ISIS), Université de Strasbourg, Strasbourg, France; Chimie ParisTech, 129667 PSL University, CNRS, Institut de Recherche de Chimie Paris, Paris, France

**Keywords:** organic molecules, transition metal complexes, rare-earth ion complexes, optica and spin coherence, qubits, quantum technologies

## Abstract

Efforts to harness quantum hardware relying on quantum mechanical principles have been steadily progressing. The search for novel material platforms that could spur the progress by providing new functionalities for solving the outstanding technological problems is however still active. Any physical property presenting two distinct energy states that can be found in a long-lived superposition state can serve as a quantum bit (qubit), the basic information processing unit in quantum technologies. Molecular systems that can feature electron and/or nuclear spin states together with optical transitions are one of the material platforms that can serve as optically addressable qubits. The attractiveness of molecular systems for quantum technologies relies on the fact that molecular structures of atomically defined nature can be obtained in endless diversity of chemical compositions. Crucially, by harnessing the molecular design protocols, the optical and spin (electronic and nuclear) properties of molecules can be tailored, aiding the design of optically addressable spin qubits and quantum sensors. In this contribution, we present a concise and collective discussion of optically addressable spin-bearing molecules – namely, organic molecules, transition metal (TM) and rare-earth ion (REI) complexes – and highlight recent results such as chemical tuning of optical and electron spin quantum coherence, optical spin initialization and readout, intramolecular quantum teleportation, optical coherent storage, and photonic-enhanced optical addressing. We envision that optically addressable spin-carrying molecules could become a scalable building block of quantum hardware for applications in the fields of quantum sensing, quantum communication and quantum computing.

## 1 Introduction

Since the utility of quantum mechanical principles, such as superposition and entanglement, for developing quantum information processing (QIP) architectures was first proposed, remarkable advances have been made. The fundamental unit of the QIP architecture is the quantum bit or qubit, which can be realized taking advantage of a physical property – for example, the quantum nature of electronic or nuclear spin degrees of freedom – presenting two stable discrete states while being able to host arbitrary superposition states. To serve as a viable platform for practical applications, qubits should satisfy a set of basic criteria proposed by DiVincenzo [1]. These include a long enough coherence time (*T*_2_) – lifetime of the superposition state – to allow for high-fidelity quantum gate operations; definitive initialization and readout mechanisms; and controllable interactions with other qubits. The reversible ability to convert flying qubits (photons) into stationary qubits (e.g. spin qubits), and vice versa, constitutes an extended criterion which is pertinent to not only quantum computation, but also communication.

A spectrum of material platforms including superconducting architectures, colour-centres in semiconducting materials, quantum dots, trapped ions, rare-earth ions (REI) doped in inorganic host lattices and molecules, have been leveraged to host qubits [2], [3], [4], [5], [6], [7]. Some systems offer advantages when a particular DiVincenzo criterion is considered but fall short in others, motivating the constant search for new material platforms that can eventually satisfy all the criteria. For instance, only some of the mentioned material platforms provide the qubit with an optical interface. The latter is however necessary to connect distant qubits and can also allow for reading out their state without needing bulky components, e.g. travelling-wave parametric amplifiers (TWPA). Optical addressing could therefore help increasing the overall number of qubits while also decreasing the size of quantum processors [8]. A classic example of an optically addressable qubit is the nitrogen-vacancy (NV^−^) centre in diamond. Due to the efficient optical spin-state initialization and readout combined with long spin *T*_2_, also at room temperature (RT), NVs have been used as fundamental building blocks for multi-qubit quantum registers [3], fault-tolerant qubit operations [9], and multi-node quantum networks [10]. NVs are also widely used for quantum sensing [11]. However, finding stable emitters among these defect centres can be challenging, as well as their integration into photonic architectures. Rare-earth ions (REIs) doped into inorganic host lattices constitute another example of optically-addressable solid-state qubit platform [2], [12]. Their major attribute is a combination of very long optical, electron- and nuclear-spin coherence times at cryogenic temperatures [13], [14], [15], [16], making them ideal systems for realizing coherent spin-photon interfaces [17]. Crucially, coherent superpositions in electron and nuclear spin transitions of REIs can be created in a fully optically manner, i.e. by leveraging a lambda scheme [16], or alternatively, using MW or RF fields [13]. Electron and nuclear spin states can also be initialized and read out optically, but photonic enhancement is required for efficiently preforming these operations at the single ion level [12] due to the weak light–matter coupling of the forbidden 4*f*-4*f* transitions.

**Figure 1:**
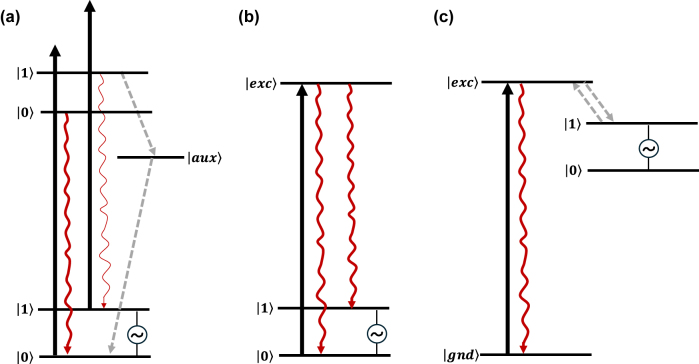
Optical initialisation and readout schemes envisioned to implement QIP protocols in molecular spin qubits. (a) Energy level scheme presenting competing spin-selective deexcitation paths allowing for spin-state initialization into $\left|0\right\rangle$ after some time of persistent optical pumping. Following coherent manipulation of the qubit states by MW pulses, the preferential radiative relaxation path towards the $\left|0\right\rangle$ state with respect to the $\left|1\right\rangle$ state can be also harnessed for qubit readout. (b) Energy level scheme allowing for a frequency-selective excitation of the spin states: the optical pumping of one of the transitions, for instance, the $\left|0\right\rangle \to \left|\mathrm{exc}\right\rangle$ , leads to population initialization into the other spin level (i.e. |1⟩). The optical selectivity in this scheme is also used for qubit-state readout. (c) Energy level scheme representing a photogenerated spin qubit, in which qubit initialization and readout relies on spin-preserving selection rules. These three schemes can be generalized to both electronic and nuclear spin states. Long spin population times are however required – at least longer than the optical excited state one – for these schemes to be implemented.

QIP protocols have been implemented in some of the mentioned solid-state platforms taking advantage of their optical addressability [2], [3], [10], [12]. However, challenges remain. For example, optically active centres are difficult to localize in a definitive manner and tuning of their physical properties relies on a limited set of protocols, e.g. the application of external electric and magnetic fields. Moreover, efficient addressing of individual qubits requires their integration with photonic cavities and microwave (MW) resonators [2], [12], which is challenging in some cases. Molecular systems could offer solutions to some of these difficulties: a remarkable intrinsic property of molecules is the fact that they can be prepared as identical copies and organized in a crystal lattice following self-assembly principles, offering enhanced design reproducibility and scalability potential with respect to the solid-state systems. Molecules can also be grafted on a range of materials platforms either covalently or non-covalently, which can be harnessed for efficient coupling to diverse photonic and MW devices [18]. Moreover, an atomistic control over the molecular structure can allow for a fine tuning of the optical and spin states [19], [20] and for designing energy level schemes supporting efficient qubit initialization, manipulation and readout protocols (Figure 1). Additionally, interactions between the qubit hosting entity and the surroundings can be engineered, paving the way for optical and spin coherence times tuning and entanglement generation [21], [22]. Consequently, efforts have been directed to leverage several classes of molecules as tunable QIP platforms [19], [23], [24], [25], [26]. In this review, we discuss light addressable molecular systems bearing electron and/or nuclear spin states – namely, organic molecules, transition metal (TM) complexes, and rare-earth ion (REI) complexes – suitable for implementing QIP protocols. Some examples present in addition potential for quantum sensing. Results such as chemical tuning of optical and spin quantum coherence, efficient optical spin initialization and readout, intramolecular quantum teleportation, optical coherent storage, and enhanced optical addressing are highlighted. It’s our opinion that molecules could be considered in a near future as scalable components of quantum hardware.

## 2 All organic chromophores as photonic quantum materials

Organic chromophore-based systems hosting electron spin states are broadly classified into two categories. In the first category, high spin-multiplicity excited states generated upon light excitation of a chromophore tethered with or without spin bearing radical is leveraged as a quantum material. This first category can be further classified into two sub-categories, namely, chromophore-based systems coupled with a radical (C-R^·^), where C and R^·^ represent the chromophore and the radical, respectively, and polycyclic aromatic systems that can undergo singlet fission (SF). The second category involves radical-ion pair states (RPs) generated because of photo-induced electron transfer (PET) in electron donor(D)- bridge(B)-acceptor(A) systems (D-B-A systems) tailored with or without an additional radical bearing entity. Systems that can undergo symmetry breaking charge transfer (SBCT) [27], [28], [29] also belong to this category, with the charge transfer involving two identical chromophores judiciously connected with each other, *vide infra*.

Single organic molecules composed of polycyclic aromatic rings are also a class of molecular systems explored for quantum technologies applications. Since they feature no spins, a discussion is not presented in this review. For more on the topic, the interested reader can consult the recent review article by Toninelli et al. [18].

### 2.1 Chromophore-radical (C-R^·^)-based systems

**Figure 2:**
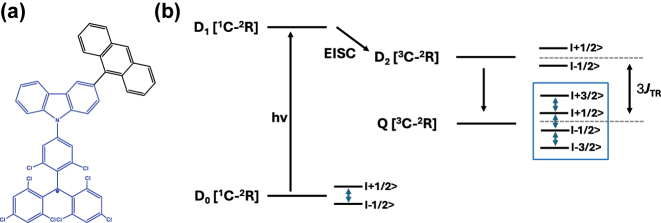
Chromophore-radical (C-R^·^)-based systems. (a) Structure of a chromophore-radical system in which optical polarization and readout of qubits have been demonstrated [33]. Radical bearing (tris(2,4,6-trichlorophenyl)methyl-carbazole) (TTM-1C_Z_) and anthracene chromophore are shown in blue and black, respectively. (b) Photophysical process involved in the C-R^·^ system leading to the formation of the high-multiplicity quartet (*Q*) state. Excitation of the C-R^·^ system into the *D*_1_ state followed by enhanced intersystem crossing (EISC) leads to the formation of *D*_2_ and *Q* states. In a strong external magnetic field, the *D*_2_ and *Q* states are separated in energy by 3*J*_TR_, where *J*_TR_ is the exchange interaction leading to polarization into the spin quartet state. Superposition states can then be created in the *Q* sublevels, represented in the figure by blue double-headed arrows, using MW pulses. Those can then be optically readout leveraging the population difference in the *Q* sublevels. Qubits based on *D*_0_ state can also be obtained by taking advantage of the polarization transfer from the *Q* state to the *D*_0_ state [34].

In the case of (C-R^·^)-based systems, a series of steps following the photoexcitation of a chromophore in the ground state lead to the formation of a high-multiplicity spin state. This state can then be polarized under an external magnetic field – termed as electron spin polarization (ESP). For such polarized states, long enough electron spin relaxation time (*T*_1_^e^) is essential for implementing QIP protocols [30], [31]. In general, excitation of a suitable organic chromophore – for example, one featuring long-lived triplet state (*T*_1_) such as fullerene (C_60_; 145 µs and C_70_; 12 ms at RT in toluene) [32] – leads first to the formation of a singlet excited state (*S*_1_) followed by conversion into a triplet state by intersystem crossing (ISC). Appending a stable radical-bearing entity with *S* = 1/2 in the chromophore skeleton enhances the rate of the singlet-to-triplet ISC, therefore the yield of chromophore-based *T*_1_ state. This mechanism is known as radical-enhanced intersystem crossing (EISC) [30]. The states involved are denoted using the notations *D*_*y*_ and *Q*, where *D* and *Q* represents overall spin multiplicity, i.e. doublet and quartet respectively, while *y* represents the ground (*y* = 0) or excited (*y* = 1 or 2) states. EISC from the *D*_1_ state leads then to the formation of doublet (*D*_2_) and quartet states (*Q*), see Figure 2 for details. Under magnetic field, a non-equilibrium spin population or polarization is observed in the *Q* state (Figure 2(b)). Qubits and higher-order qubits – qu*d*its – can then be accessed, and quantum superposition states can be created using MW excitation. The optical readout is carried out by monitoring the luminescence and absorption of these excited states produced by exchange coupling [33]. In these systems, qubit states based on the stable radical-based *D*_0_ state can be also realized taking advantage of polarization transfer from the high-multiplicity excited state to the ground *D*_0_ state (Figure 2(b)). In essence, two different types of qubits based on the *Q* and *D* states can be obtained in the same C-R^·^ system.

Table 1 provides a representative compilation of *Q*- and *D*-based electronic coherence time (*T*_2_^e^) values. The impetus for harnessing the utility of the C-R^·^ systems as quantum materials are based on time-resolved electron spin resonance (Tr-ESR) studies [35]. Those demonstrate the presence of spin-polarized excited quintet states in fullerene- (C_60_) [36], porphyrin- [37] and anthracene-based systems [38] tethered with nitroxide-based radicals and transition metalloporphyrins [39], [40], [41]. Subsequently, it has been demonstrated that the rates, sign, and magnitude of polarization transfer to the ground state can be tuned by changing the position of a radical bearing group on the chromophore [34], and by engineering the distance between the radical and chromophore [42]. In a seminal study dealing with a PDI-TEMPO based, (PDI and TEMPO stand for 1,6,7,12-tetra(4-*tert*-butylphenoxy)perylene-3,4:9,10-bis(dicarboximide, and 2,2,6,6-tetramethylpiperidine-1-oxyl, respectively) C-R^·^ system, Wasielewski, Richert, and co-workers demonstrated that photogenerated quartets in a PDI-based C-R^·^ system can be used to host electron spin qubits with *T*_2_^e^ = 1.8 μs at 80 K [20] (Entry 1; Table 1). They have also demonstrated that higher-order qu*d*its can be obtained by leveraging electron-nuclear spin hyperfine interactions. Later studies dealing with PDI-based C-R^·^ systems substantiated that spin coherence times (*T*_2_^*n*^) in the µs regime can be obtained even at RT [43] (Entry 2; Table 1). Moreover, spin-polarization from the quintet state can be transferred to the radical-based doublet state [44] (Entry 3; Table 1), rendering access to a polarized spin qubit involving the ground *D*_0_ state. In the examples discussed before, optical readout of qubit states is however inhibited by the non-luminescent nature of the radical bearing entities [45]. By designing a C-R^·^ system in which both chromophore (anthracene) and radical bearing entities (tris(2,4,6-trichlorophenyl)methyl-carbazole) are luminescent (Figure 2; entry 4, Table 1), Friend, Evans, and co-workers demonstrated that efficient readout of qubit states can be achieved [33], [46]. Optical polarization and readout are mediated by the close energetic proximity between the triplet state of anthracene and doublet excited state of the carbazole-based radical in the C-R^·^ system. In this system, excited states combining high-multiplicity with near unity quantum yield can be obtained.

**Table 1:** Electron spin coherence times of chromophore-radical (C-R^·^)-based molecular systems.

Entry	Molecule	Medium	*T*_2_^e^	Temperature	Ref.
1	PDI-TEMPO **Figure:** 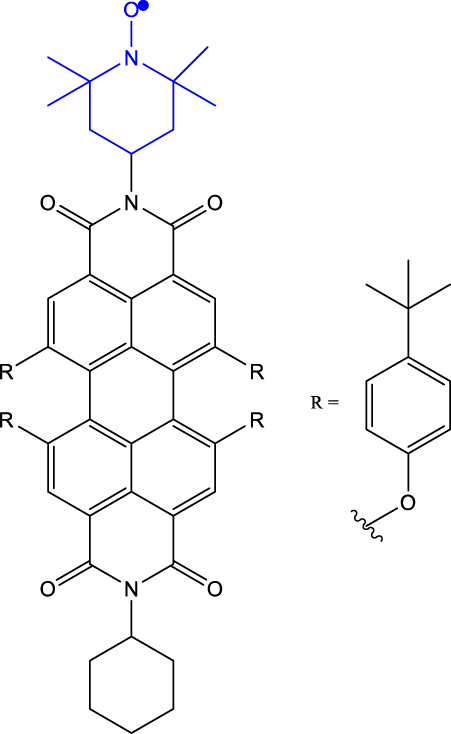	Frozen toluene solution	1.8 µs (*Q*)	80 K	[20]
2	PDI-TEMPO-*d*_17_-^15^ *N* **Figure:** 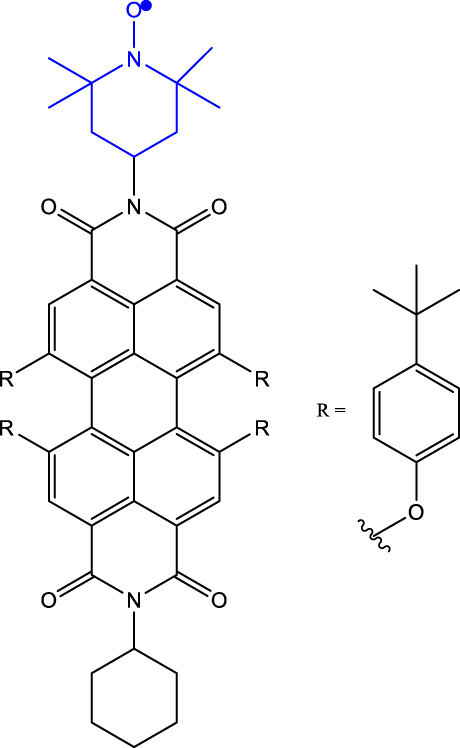	Polymer matrix	1.1 µs (*D*_0_) 0.7 µs (*Q*)	300 K	[43]
		Frozen toluene solution	4.4 µs (*D*_0_) 2.6 µs (*Q*)	80 K	
3	tpPDI-BDPA-*d*_16_^*a*^ **Figure:** 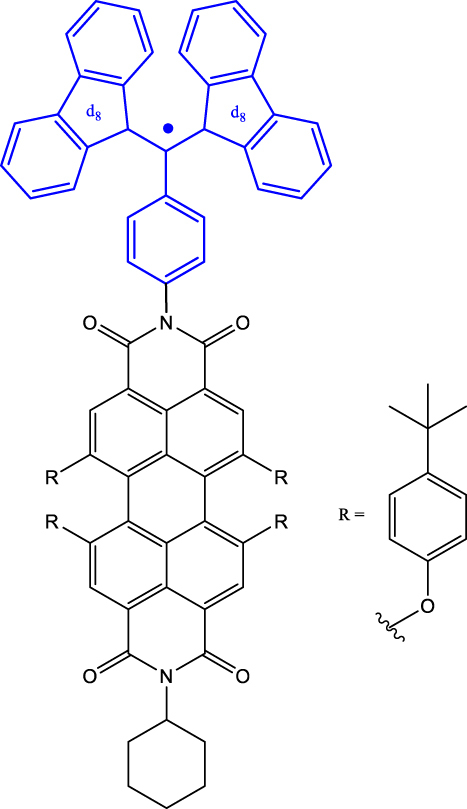	2-methyl tetrahydro-furan (mTHF) glassy matrix	2.1 µs (*D*_0_) 2.8 µs (*Q*)	85 K	[44]
4	Anthracene-(tris(2,4,6-trichlorophenyl)methyl-carbazole) (TTM-1Cz-An) **Figure:** 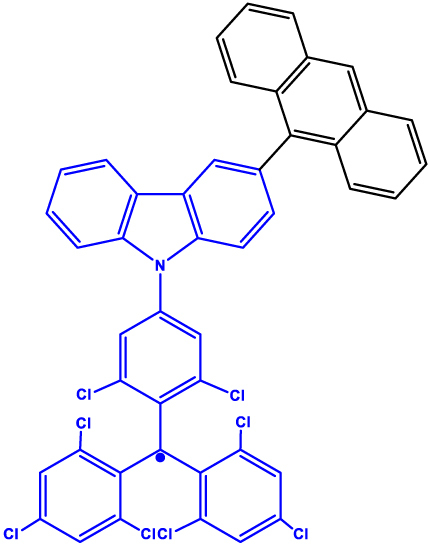	Frozen toluene solution and PMMA matrix	10 µs (*Q*)	295 K	[33]
5	p-*d*_*5*_-PDI-CHAMPO-*d*_*25*_ derivatives^*b*^ **Figure:** 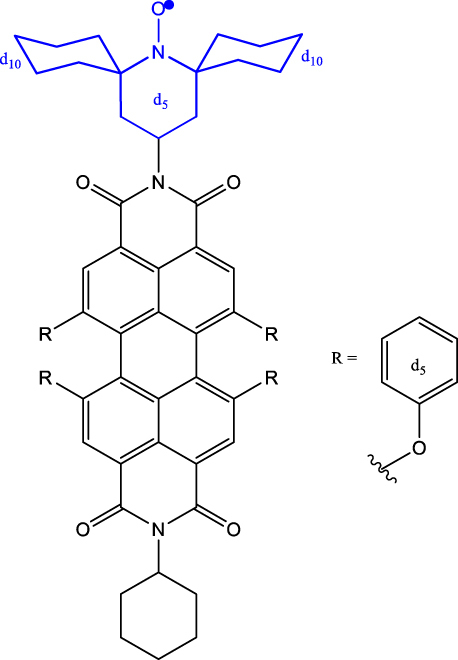	Frozen toluene solution	9.1 µs (*D*_0_) 4.2 μs (*Q*)	85 K	[48]
6	PDI-biph-tetrathiaryltrityl^*c*^ **Figure:** 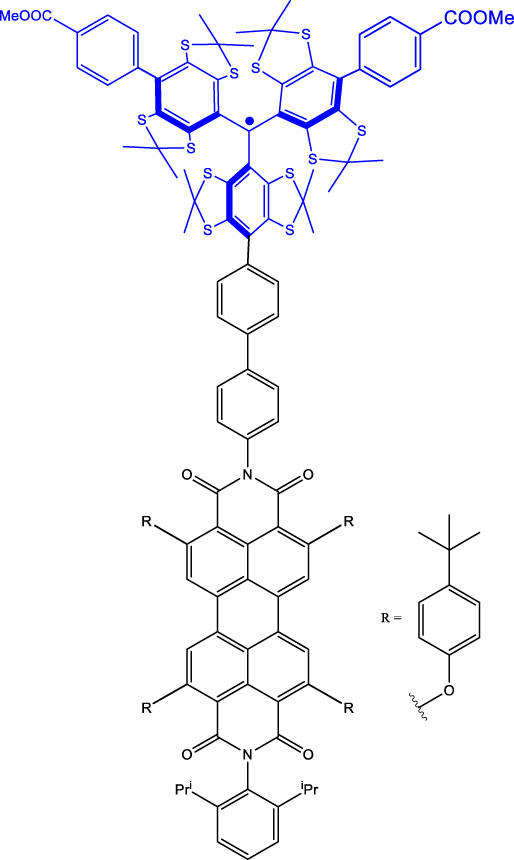	Frozen toluene solution	4.7 µs (*D*_0_) 3 μs (*Q*)	80 K	[47]

^a^BDPA stands for 3-bisdiphenylen-2-phenylallyl. ^b^CHAMPO: methyl groups of TEMPO are replaced by spirocyclohexyl groups. ^c^One among the three C-R^·^ systems featuring long coherence time is shown; see ref. [47] for two other examples.

As discussed before, chemistry can be leveraged to tune the nature of polarization transfer, exchange coupling between chromophore and radical, and electron spin coherence times. *T*_2_^e^ enhancement following isotope substitution and chemical structure modifications have been demonstrated for PDI-TEMPO systems [43], [48]. In particular, methyl group tunnelling in TEMPO radical contributes to decoherence in PDI-TEMPO systems. It has been demonstrated that by replacing methyl groups with cyclohexyl groups – TEMPO versus CHAMPO in Table 1 – *T*_2_^e^ of chromophore-centred Q and radical-centred D states can be increased (Entry 5; Table 1). The chemical nature of radical bearing system also influences the coherence time. A comparison between the *T*_2_^e^ values reported for PDI-based chromophore systems featuring TEMPO, BDPA, and trityl radicals reveals that the PDI-trityl (Entry 6; Table 1) system shows longer *T*_2_^e^ relative to its two other counterparts [47]. Such enhancement is due to the mitigation of hyperfine interactions in the trityl radical relative to the TEMPO and BDPA systems. It has also been reported that the medium – polymer matrix versus frozen toluene solution – in which C-R^·^ systems are diluted and concentration of the systems, directly modulate the *T*_2_^e^ value [49]. The above points emphasize the fact that several criteria need to be optimized to progress toward implementing QIP schemes using these molecular systems.

### 2.2 Polycyclic aromatic systems capable of undergoing singlet fission

**Figure 3:**
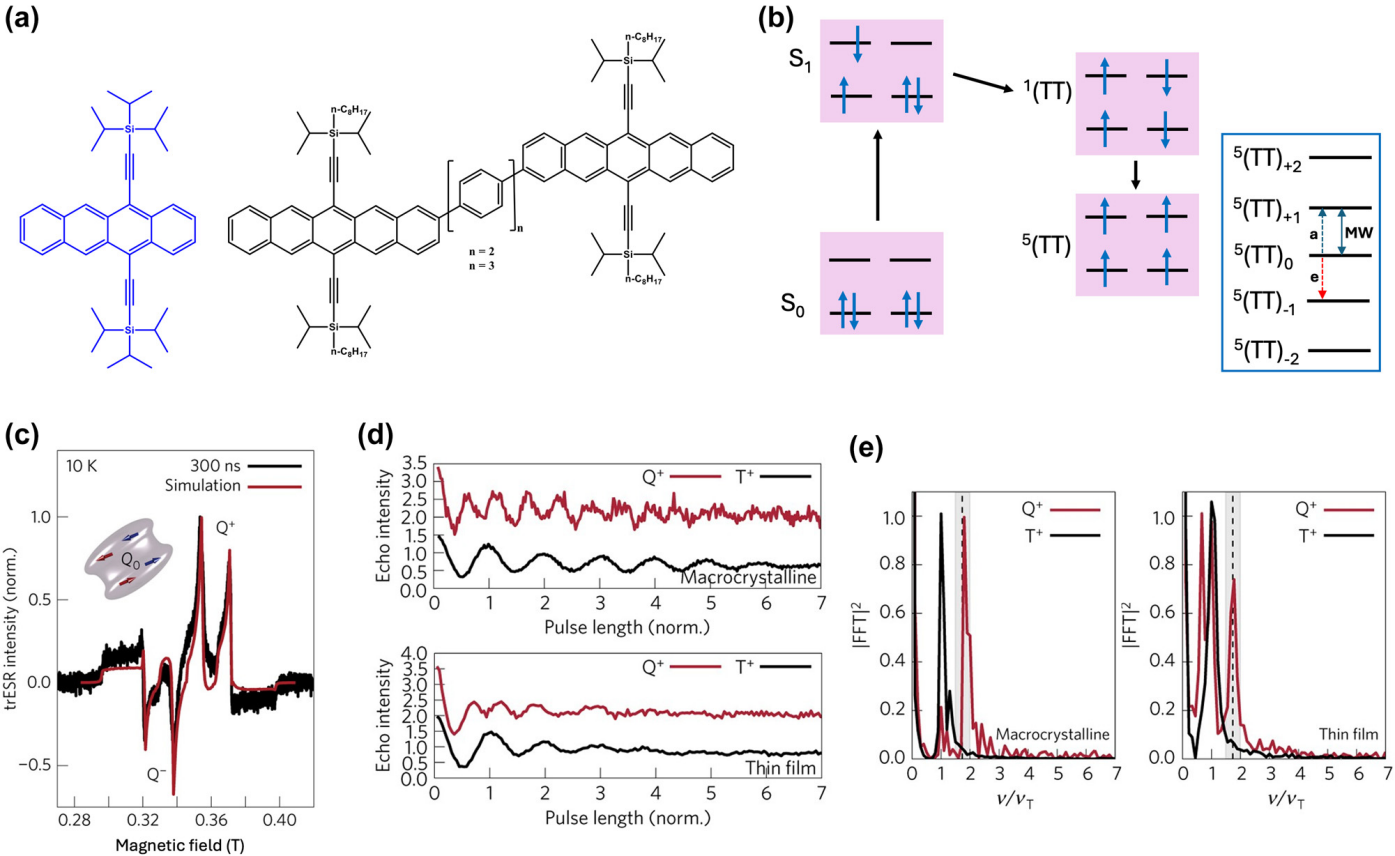
High spin-multiplicity excited states generated from singlet fission (SF) for QIP applications. (a) Molecular structures of TIPS-tetracene and pentacene dimer in which SF-mediated generation of entangled ^5^(TT) pair have been demonstrated. (b) Mechanism of ^5^(TT) pair formation. Excitation of singlet pair leads to the formation of singlet excited state that decays into ^1^(TT) and then ^5^(TT) states. In the presence of magnetic field, electronic spins are polarized in the ^5^(TT)_0_ state. By applying a MW pulse of suitable frequency, coherence between the ^5^(TT)_0_ and ^5^(TT)_1_ states, for example, can be created. The absorption **a** and emission **e** from the ^5^(TT)_0_ state can then be used as readout. (c) Transient ESR spectrum of TIPS-tetracene at 10 demonstrating the presence of the ^5^(TT) quintet pair. The outer components are from the triplet state associated with single TIPS-tetracene molecules. The *Q*− and *Q*+ terms represent emission and absorption from the ^5^(TT)_0_ state. (d) Rabi oscillations associated with the TIPS-tetracene in thin-film and microcrystalline states. The notations *Q* ^+^ and *T* ^+^ represent oscillations arising from the ^5^(TT) quintet and triplet states, respectively. (e) Fourier-transform of the oscillations shown in figure (d). Oscillations from the ^5^(TT) quintet state have higher frequency than those from the triplet state, serving as the unambiguous proof for the presence of the high spin multiplicity quintet state. Figures (c), (d), and (e) are reproduced with permission from ref. [50].

Entangled triplet excitons of high spin-multiplicity obtained as a consequence of SF have also been demonstrated as qubits. Strong exchange coupling between triplet pairs (Figure 3) gives rise to an exchange coupled quintet state (^5^TT; S = 2). Similar to the C-R^·^ case, the application of magnetic field brings the spin-population out of equilibrium, ultimately leading to spin polarization. Notably, the coupling of two triplet states producing an entangled pair of high multiplicity represents the fact that higher-order qu*d*its, involving five states, can be obtained from SF relative to the three states associated with a triplet state located on a single-molecule [50], [51], [52], [53]. Till date, the utility of this ^5^TT has been demonstrated in pentacene- and tetracene-based systems (Figure 3(a)). Two seminal studies unambiguously elucidated the presence of a ^5^TT state by studying tetracene- [50] and pentacene-based [51] molecular systems at low temperatures. The later study demonstrates SF intramolecularly in pentacene as against the interdimer tetracene pair studied in the former case. Intermolecular ^5^TT pair lifetime of 3 µs and *T*_2_^e^ of 0.87 µs have been obtained at 10 K [50] for TIPS-tetracene where TIPS stands for triisopropylsilylethynyl group) = (Entry 1; Table 2). Although these first studies dealing with the ^5^TT pair focus more on elucidating the formation and dynamics of the ^5^TT pair, the observation of Rabi oscillations elucidate the utility of these intramolecularly generated ^5^TT pairs for QIP [50], [51].

**Table 2:** Electron spin coherence times of molecular systems hosting ^5^TT state derived from SF.

Entry	Molecule	Medium	*T*_2_^e^	Temperature	Ref.
1	Triisopropylsilylethynyl (TIPS)-tetracene **Figure:** 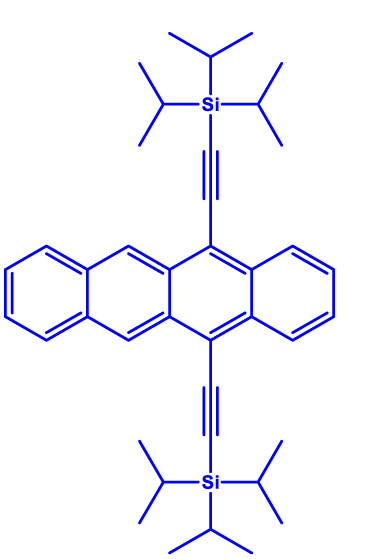	Thin-film and microcrystals	∼1 µs	10 K	[50]
2	tricyclohexylsilylethynyl (TCHS)-tetracene **Figure:** 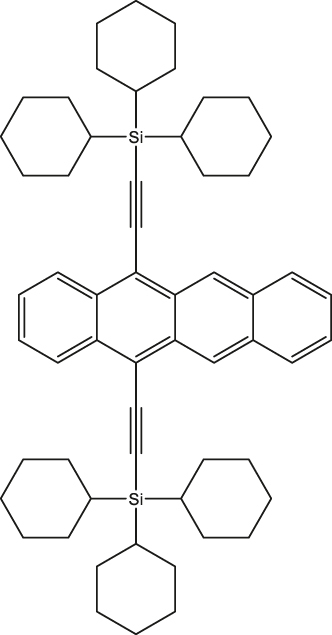	Single crystals	3/10 µs	10 K	[52]
3	TIPS-bipentacene **Figure:** 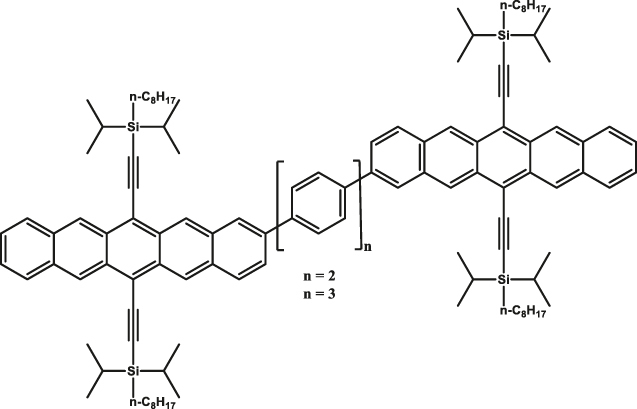	Intramolecular dimer in dilute 2-methyltetrahydrofuran (mTHF) glass	1.4 µs	75 K	[53]
4	4,4′-(pentacene-6,13-diyl)dibenzoic acid (PDBA) **Figure:** 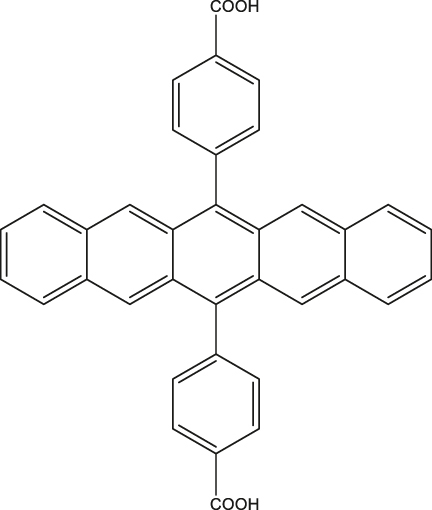	PDBA-based metal-organic framework	0.150 µs	300 K	[54]

In a quest to optimize the dynamics of ^5^TT pair formation, Wasielewski and co-workers designed a new tetracene derivative – TCHS-tetracene, where TCHS is tricyclohexylsilylethynyl – and showed that by engineering the packing in the crystal lattice via molecular design, coherence times can be improved [52]. In particular, *T*_2_^e^ = 3 µs is observed at 10 K (Entry 1; Table 2), and the authors have also demonstrated that the *T*_2_^e^ can be further increased to 10 µs by employing dynamical decoupling (Entry 2; Table 2) [52]. A recent study dealing with a covalently linked dimer elucidates that a *T*_2_^e^ in the order of 1.4 µs can be obtained at 40 K [53] (Entry 1; Table 3). The utility of chemistry in generating pentacene-based ^5^TT systems for QIP applications have been further demonstrated in a recent study dealing with a pentacene-based MOF. Due to the rigid nature of the MOF backbone, mechanisms such as triplet-triplet annihilation and exciton diffusion have been mitigated resulting in the observation of 150 ns coherence time at RT [54] (Entry 4; Table 2).

**Table 3:** Electron spin coherence times of D-B-A molecular systems.

Entry	Molecule	Medium	*T*_2_^e^	Temperature	Ref.
1	Tetrathiafulvalene (TTF; donor)-1,8-dicarboximide (ANI; bridge/chromophore)-pyromellitimide (PI; acceptor) **Figure:** 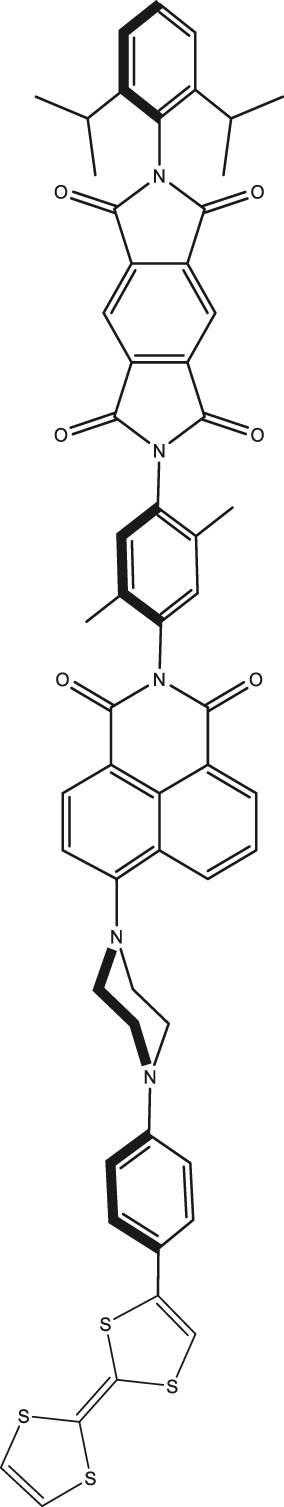	4-cyano-4′-(*n*-pentyl)-biphenyl (5CB)^a^	1.8 µs	85 K	[55]
2	2,6,6-tetramethylbenzo[1,2-*d*:4,5-*d*′]bis([1, 3]dioxole) (donor)-4-aminonaphthalene-1,8-imide (acceptor/chromophore) and α,γ-bisdiphenylene-β-phenylallyl (radical; partially deuterated)^b^ **Figure:** 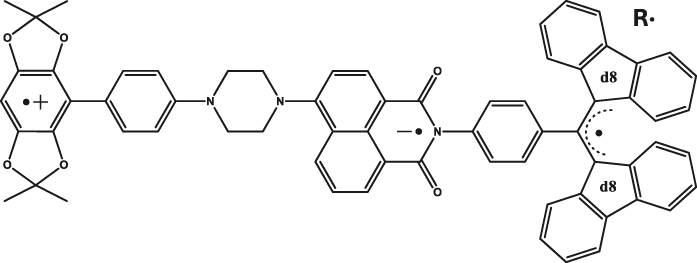	Butryonitrile glass	0.89 µs (D^·+^) 1.16 µs (R^·^)	85 K	[22]
3	Pyrene (Py)-Napthalene dianhydride (A) **Figure:** 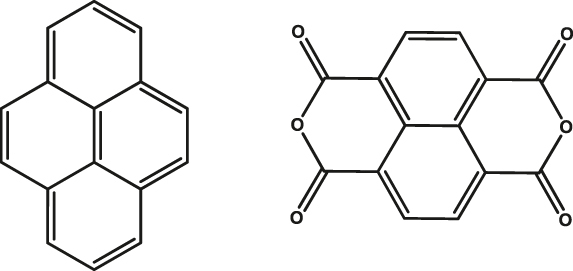	Single-crystal	7.1 µs	20 K	[56]
4	Napthalenediimide (NDA; acceptor)-DNA (bridge)-tetrathiofulvalene (TTF; donor) **Figure:** 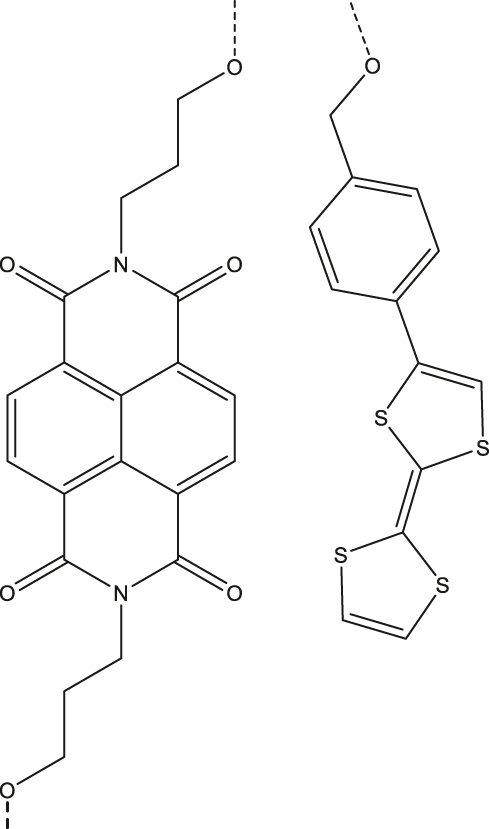	Deuterated buffer solution	4 µs	85 K	[57]
5	5,12-diazatetracene (DAT) chromophore in a metal-organic framework (MOF)^c^ **Figure:** 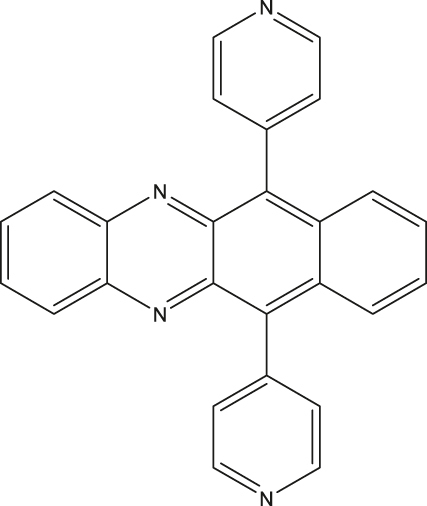	MOF dispersed in paraffin	0.98 µs	RT	[31], [58]

^a^Nematic liquid crystal. ^b^Structure shown in Figure 4(a). ^c^SBCT-active, see [31] for two additional examples.

### 2.3 D-B-A systems

Within the category of electron donor (D) - bridge (B) - acceptor (A) pairs, photogenerated qubits have also been demonstrated [59], [60]. In a typical D-B-A system, electron-rich and -deficient fragments are connected via a bridge (B). Molecular distance engineering between D and A fragments can be leveraged to modulate the interactions between the fragments. Optical excitation of one of the components in a D-B-A system leads to the formation of a charge-separated state composed of an excited state radical-ion-pair (for example, D^·+^-B-A^·−^) coupled via spin-exchange and dipolar interactions. Those are often referred to as a spin-qubit pairs (SQPs) in the context of QIP. In SQPs, state polarization is achieved under external magnetic fields and the resultant absorptive and emissive transitions can be probed by performing time-resolved (pulsed) ESR studies. The mechanism of optical spin polarization in D-B-A systems is described in details Figure 3(b).

**Figure 4:**
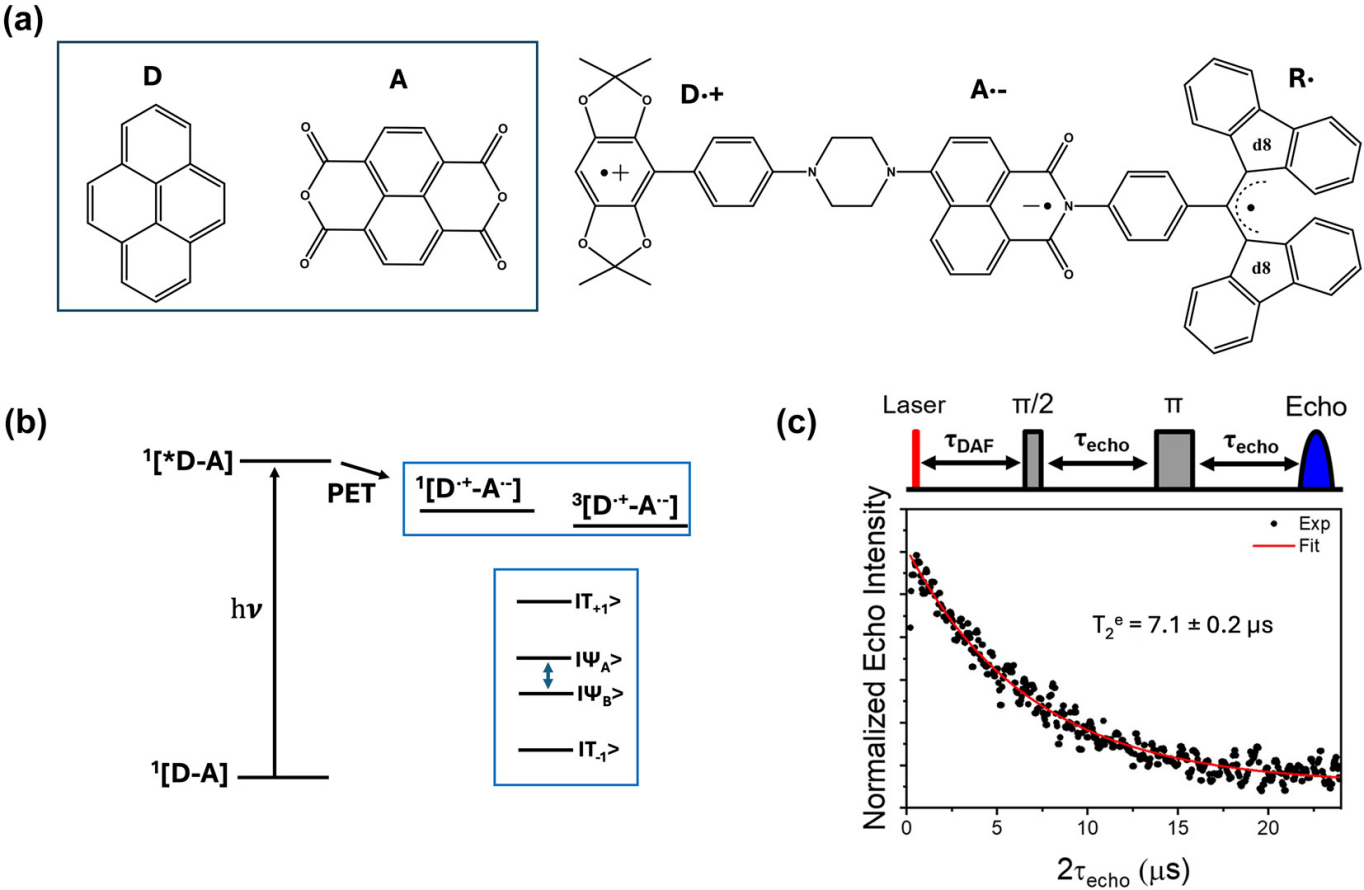
Electron donor (D)-bridge (B)-acceptor (A) systems as photoinduced spin qubits. (a) Structures of co-crystallized and covalently linked D-A systems. The notations D^·+^, A^·−^ and R^·^ in the top structure depicts donor-based cationic radical, acceptor-based anionic radical, and the appended stable organic radical, respectively. (b) Mechanism of optical spin polarization in D-B-A systems. Excitation of a D-B-A system in the ground state leads to the formation of the singlet excited state – ^1^[*D-B-A], with the donor fragment excited in this specific case. Photo-induced electron transfer (PET) from the donor to the acceptor leads to the formation of the charge-separated state. Due to the spin conserving nature of the PET process, a singlet state – ^1^[D^·+^-B-A^·−^] – is formed initially. A subsequent spin-flip leads to the formation of the triplet spin-pair. In a static magnetic field that is relatively large compared to the spin–spin exchange (J) and dipolar (D) interactions, mixing between the ^1^[D^·+^-A^·−^] and ^3^[D^·+^-A^·−^] states produces hybrid electronic levels, as shown in the blue box [61]. Due to the singlet character of the ^1^[D^·+^-B-A^·−^] state, the Ψ_A_ and Ψ_B_ levels are initially populated. By using appropriate MW pulses and leveraging the mentioned spin-polarization into the Ψ_A,B_ states, coherence superposition between the mixed singlet and triplet levels can be created. Addition of a radical bearing entity has been exploited to generate higher order qubits, as in the structure shown in (a, right). (c) To circumvent line-broadening and the consequent qubit addressability issue, co-crystallized D-A systems have been proposed (a, left). Spatially oriented triplet excitons in such systems resulted in the observation of about 7.1 µs coherence lifetime at 20 K. In the top part, the pulse sequence used to determine the coherence lifetime is shown [56]. Figure (c) is reproduced with permission from [56].

Typically, *T*_2_^e^ values in the order of several microseconds have been demonstrated for a range of D-B-A systems – namely, molecular systems tailored with or without stable organic radicals [59], D-A entities tethered with DNA hairpins [61], [57] (Entry 4; Table 3), and organic dye functionalized quantum dot (QD) [62]. In the last case, electron transfer from the dye to the QD generates the SQP. The microsecond-long *T*_2_^e^ of an entangled spin-pair residing on a D-B-A skeleton (Entry 1; Table 3) was leveraged to demonstrate two qubit gate operations [55]. Addition of a stable radical bearing entity into a D-B-A framework enables the creation of three-spin quantum systems – D^·+^-B-A^·−^-R^·^ (Figure 4(a)-right and Entry 2; Table 3) – in which spin polarization can be transferred from one fragment to another fragment, enabling quantum teleportation in the MW regime at nm distances. This has been demonstrated, elucidating short-range quantum interconnectors based on molecules can be realized [22]. Hyperfine interactions between the electron and nuclear spins are one of the factors driving decoherence in D-B-A systems. *T*_2_^e^ can however be enhanced by mitigating the hyperfine interaction by isotopically enriching molecular skeletons with nuclei that bearing small magnetic moments – for example, replacing hydrogen with deuterium [20], [44], [59]. One general limitation of the molecular systems is the random orientation of the molecules – therefore the g-tensor – with respect to an external magnetic field. This contributes to line broadening of the EPR transitions, typically in the range of GHz, making selective addressing of individual transitions challenging. In this respect, it has been recently demonstrated that spatially oriented triplet excitons can be realized in a co-crystal composed of donor (pyrene) and acceptor (naphthalene dianhydride) entities (Figure 4(a)-left and Entry 3; Table 3). For the system, selective addressing of the spin levels has been reported, as well as *T*_2_^e^ of 7.1 μs at 20 K [56].

Electron-transfer-mediated creation of SQPs can also be generated in molecular systems that can undergo symmetry breaking charge transfer (SBCT). Unlike in the cases of D-B-A systems discussed above, in which electron deficient and rich fragments are involved in the charge transfer process, identical molecular fragments orthogonally connected in a dimer are involved in the SBCT process. Upon light absorption, one of the fragments in an orthogonally connected dimer is excited and a locally excited state is produced. Differences in the local solvent interactions around the dimer creates the driving force required for charge separation, thereby charge separation and stabilization of the SQP is facilitated [28]. Efficient spin polarization, polarization transfer from the triplet to persistent radicals, and long *T*_1_^e^ and *T*_2_^e^ of polarized radicals at RT have been demonstrated for SBCT-active metal-organic frameworks (MOF) composed of 5,12-diazatetracene (DAT) chromophore [31], [58] (Entry 5; Table 3). Finally, an interesting approach to increase the utility of D-B-A systems for QIP is the use of a chiral bridge [63]. By harnessing chirality-induced spin selectivity (CISS) effects, strong spin polarization can be achieved, setting the basis for high-fidelity qubit initialization, manipulation and single-spin readout. Some examples of chiral bridges in which strong spin polarization (>90 %) has been reported are supramolecular wires, 2D chiral hybrid lead-iodide perovskites or chiral hybrid copper halides [63].

## 3 Metal complexes as optically addressable spin qubits

**Figure 5:**
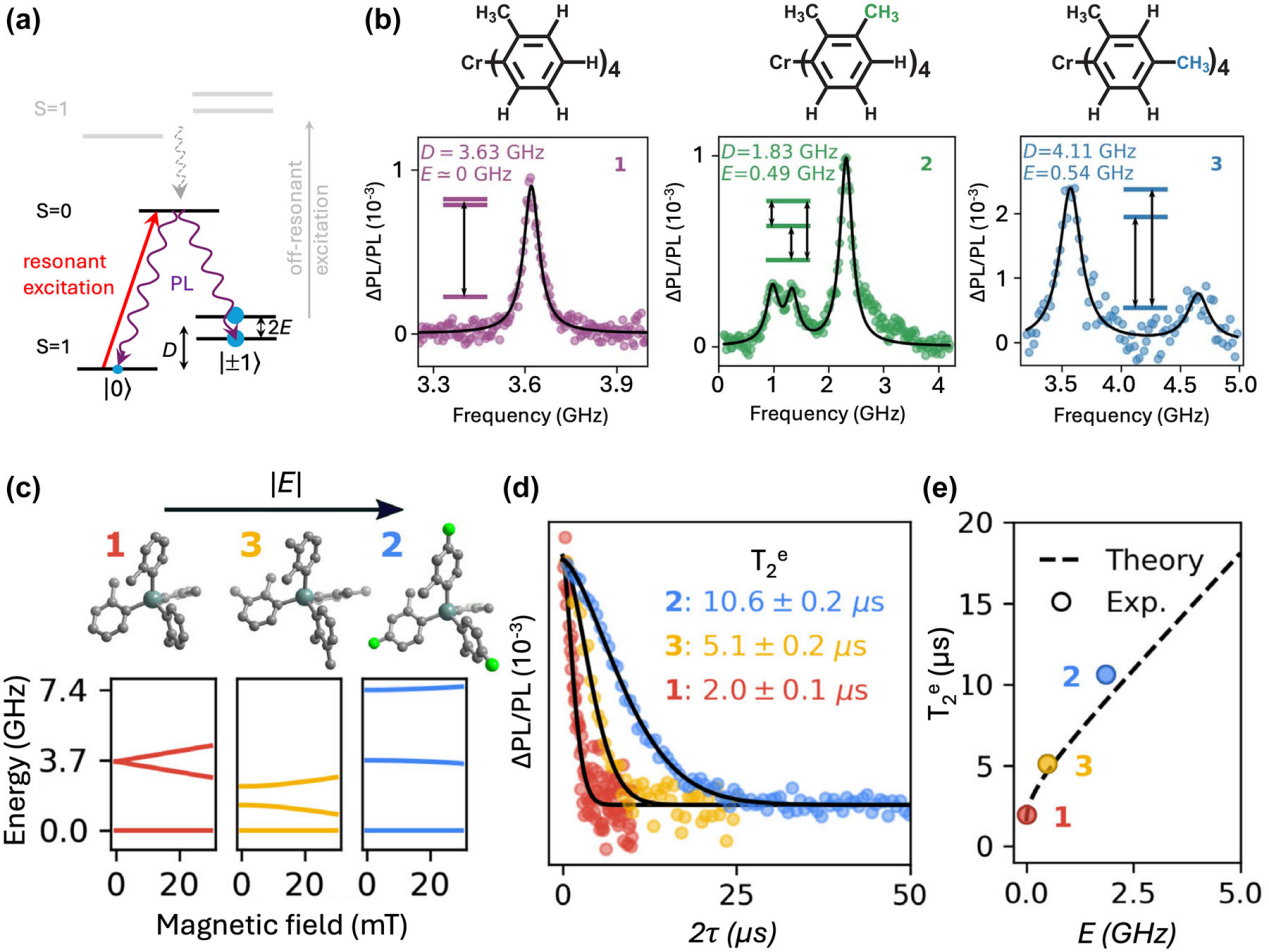
Cr^4+^-based complexes as optically addressable qubits. (a). Energy-level structure of Cr^4+^-based complexes showing spin-state selective excitation and emission between the ground *S* = 1 and first-excited *S* = 0 states. *D* and *E* represent the axial and transverse zero-field splitting parameters, respectively. (b). Continuous-wave optically detected magnetic resonance (CW-ODMR) spectra of three Cr^4+^ complexes – referred to as 1, 2, and 3, in the figure. These three complexes differ by the number and position of methyl groups on the ligand skeleton. This subtle structural difference gives rise to clearly distinct zero-field splitting and its evolution under magnetic field, with direct impact on the achievable *T*_2_^e^. The figure is adapted with permission from ref. [65]. (c). Molecular structures forming the Sn-based host matrices 1 (red), 2 (yellow), and 3 (blue) used to embed complex 1, and their simulated spin energy levels as a function of the magnetic field. (d). Optically detected Hahn-echo traces for single crystals of 1, 2, and 3 containing complex 1, at zero magnetic field. (e). Zero-field *T*_2_^e^ as a function of the transverse zero-field splitting *E* along with the theoretical dependence calculated from first-principles cluster-correlation expansion (CCE) methods. Adapted from ref. [21].

The group of elements known as transition metals (TM) occurs in the fourth period of the periodic table. The incomplete nature of the 3d shell results in a set of electronic energy levels with energies dependent on the strength and symmetry of the ligand field. Under certain ligand field symmetries, TM complexes featuring electronic spin triplet (*S* = 1) ground state and spectrally narrow metal-centered optical transitions can be obtained. Such properties make TM complexes a very attractive platform for quantum technologies, sharing important similarities with other optically active defects [64] (Figure 1(a)). *Fataftah and Freedman* [19] set the main molecular design criteria for TM complexes to be used as optically-addressable electron spin qubits: a zero-field splitting (D) within the EPR addressable range and an electronic structure in which the first excited state is a spin singlet (*S* = 0). This second criterion allows for either a non-resonant excitation through a spin-selective competitive deexcitation scheme involving intersystem crossing (ISC), or alternatively, a resonant and frequency-selective excitation of the ground-state spin levels for initialization and readout of the qubit states (Figure 5(a)). A final and fundamental criterion for TM complexes, shared by all discussed platforms to be eligible qubits, is presenting long enough electronic spin population time (*T*_1_^e^) and coherence time (*T*_2_^e^).

Leveraging the strong sensitivity of TM ions to the ligand field environment and the power of coordination chemistry for fine-tuning the later, TM complexes fulfilling some – or all – the previously mentioned criteria, have been reported. In particular, it has been demonstrated that a rational design of the spin–orbit coupling, ligand field and symmetry can lead to EPR and optical addressability in Cr^3+^, Cr^4+^, V^3+^, V^4+^ and Ni^2+^ complexes [21], [65], [66], [67], [68], [69]. The salient results are summarized in the following sub-sections.

### 3.1 Cr^3+^ and Cr^4+^ complexes

The combination of sub-GHz optical linewidths with long-lived electronic spin states in TM complexes was first reported during the late eighties and nineties using the spectroscopic technique known as persistent spectral hole burning (SHB) [69]. These early studies were mostly focused on Cr^3+^-based complexes – for example, [Cr(en)_3_]^3+^ – showing optical homogeneous linewidths (Γ_h_^opt^) of the order of 150 MHz for the R1 line of Cr^3+^ at liquid helium temperature [70]. This corresponds to an optical coherence time (*T*_2_^opt^) of about 2 ns, according to the relation Γ_h_^opt^ = 1/*πT*_2_^opt^. Alternatives to reduce this broadening, and consequently improve *T*_2_^opt^, include approaches such as diluting the complexes (≤0.001 %) in host lattices and leveraging exchange interactions to minimize magnetic noise. For example, intramolecular anti-ferromagnetic coupling in binuclear Cr^3+^ complexes results in an *S* = 0 ground state leading to the nullification of electron-spin–based dipolar interactions [71]. The possibility of modulating the energy-level structure and the optical and electron spin coherence times through complex and host design has also been reported [69]. In particular, by combining several strategies, narrow optical homogeneous linewidths of 40 MHz were obtained in single crystals of perprotonated and partially deuterated versions of the binuclear [LCr(III)(-OH)_3_ – Cr(III)L](ClO_4_)_3_·H_2_O (L = 1,4,7-trimethyl-1,4,7-triazacyclo- nonane) complex [72].

These pioneering studies, many of them authored by Riesen and coworkers [69], [70], [72], [73], remain, to the best of our knowledge, as the state-of-the-art in terms of Γ_h_^opt^ in TM complexes. However, correlations between the molecular design and qubit figures of merit – for example *T*_2_^e^ or the ability to initialize the qubit, have not been considered in these studies. In contrast, such correlations have been elucidated for Cr^4+^-based complexes [21], [65].

To meet optically addressable electron spin qubit design criteria, Bayliss and co-authors designed three Cr^4+^ complexes coordinated by strong-field aryl ligands in a pseudo-tetrahedral configuration. This highly symmetrical environment yields a ground state spin triplet (*S* = 1) with small zero-field splitting – 1.9–4.2 GHz – while the strong-field is leveraged to obtain the *S* = 0 state as the lowest-lying electronic excited state (Figure 5(a)). Optical characteristics of three complexes differing in the number and position of the methyl groups (Figure 5(b)) were studied by diluting them in a suitable crystalline host lattice. The minor structural difference results in distinctive ground-state axial (*D*) and transverse (*E*) zero-field splitting parameters and optical transition energies for the three complexes. Utility of the complexes as optically addressable electron spin qubits is elucidated by the demonstrations of optical spin state polarization (14 %) and readout under resonant excitation to the excited (*S* = 0) state, and spin-state manipulations using MW excitation (Figure 5(a)). In particular, optically-detected Rabi oscillations and a *T*_2_^e^ of 640 ns at 2 mT and 4 K [65] have been observed. This relatively short coherence time was improved to about 10 μs in a subsequent work, harnessing the energy-level-structure tunability through the host environment modification [21]. The engineering of the host crystal structure allows for designing magnetically insensitive – clock – electron spin transitions [16]. Complex 1 in Figure 5(b) was diluted down to 0.5 %–1 % in non-isostructural Sn-based hosts, giving rise to a non-zero transverse zero-field splitting (*E* ≠ 0) due to symmetry breaking with respect to the isostructural host ‘1’, as well as to lower (host ‘3’), or negligible (host ‘2’) transition energy variation under magnetic field (Figure 5(c)). A significant improvement in *T*_2_^e^ was in particular reported for the non-isostructural host ‘2’, reaching 10 μs at 4 K [21] (Entry 1; Table 4).

**Table 4:** Optically addressable electronic qubits and quantum sensor candidates based on TM complexes.

Entry	Compound	Medium	*T*_2_^e^ or *T*_m_^a^	Γ_inh_^opt^ ^b^	Wavelength	Ref.
1	Cr(IV)(o-tolyl)_4_^a^ **Figure:** 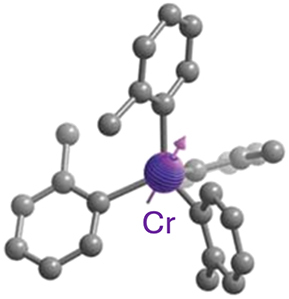	Sn(IV)(4-fluoro-2-methylphenyl)_4_ crystal	*T*_2_ = 10 μs at 4 K	53.2 GHz at 4 K	1,016 nm	[21]
2	(C_6_F_5_)_3_trenVCN^t^Bu **Figure:** 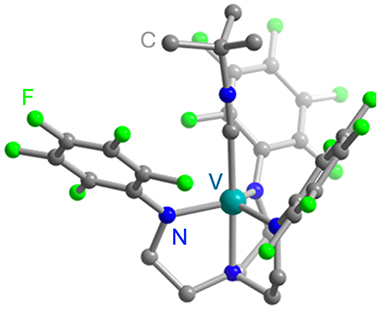	Diluted into (C_6_F_5_)_3_trenGaCN^t^Bu analoge matrix	*T*_m_ = 427 ns at 5 K and 6.55 T	∼90 GHz at 4 K	1,237 nm	[66]
3	[(VO)_2_(bdhb)_2_] **Figure:** 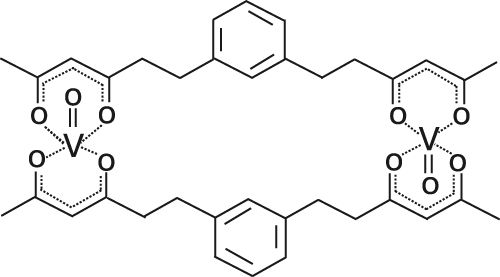	frozen toluene-*d*_8_/CD_2_Cl_2_ matrix	*T*_m_ = 13 μs at 10 K and 0.34 mT	Not reported	Not reported	[74]
4	[Ni(phen)_3_](BF_4_)_2_] **Figure:** 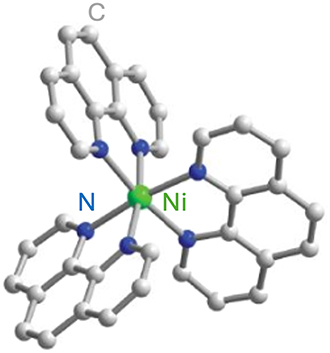	1 mM solutions in 1:1 H_2_O:glycerol	*T*_m_ = 250 ns at 20 K and 1.53 T	∼100 THz at 80 K	940 nm	[67]
5	Ni(ttcn)_2_](BF_4_)_2_ **Figure:** 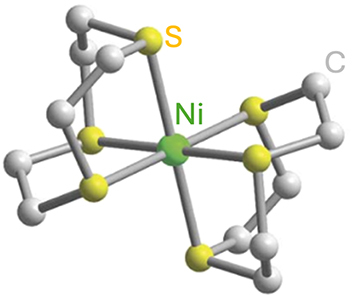	diluted into [Zn(ttcn)_2_](BF_4_)_2_ analogue matrix	*T*_m_ ∼ 400 ns at 12 K and 0.25 T	Not reported	782 nm	[68]

^a^ *T*_m_ stands for phase memory and encompasses all processes that contribute to decoherence, including *T*_2_^e^ and the inhomogeneous dephasing time *T*_2_^e^ *. ^b^Γ_inh_ stands for optical inhomogeneous linewidth.

### 3.2 Ni^2+^, V^3+^ and V^4+^ complexes

**Figure 6:**
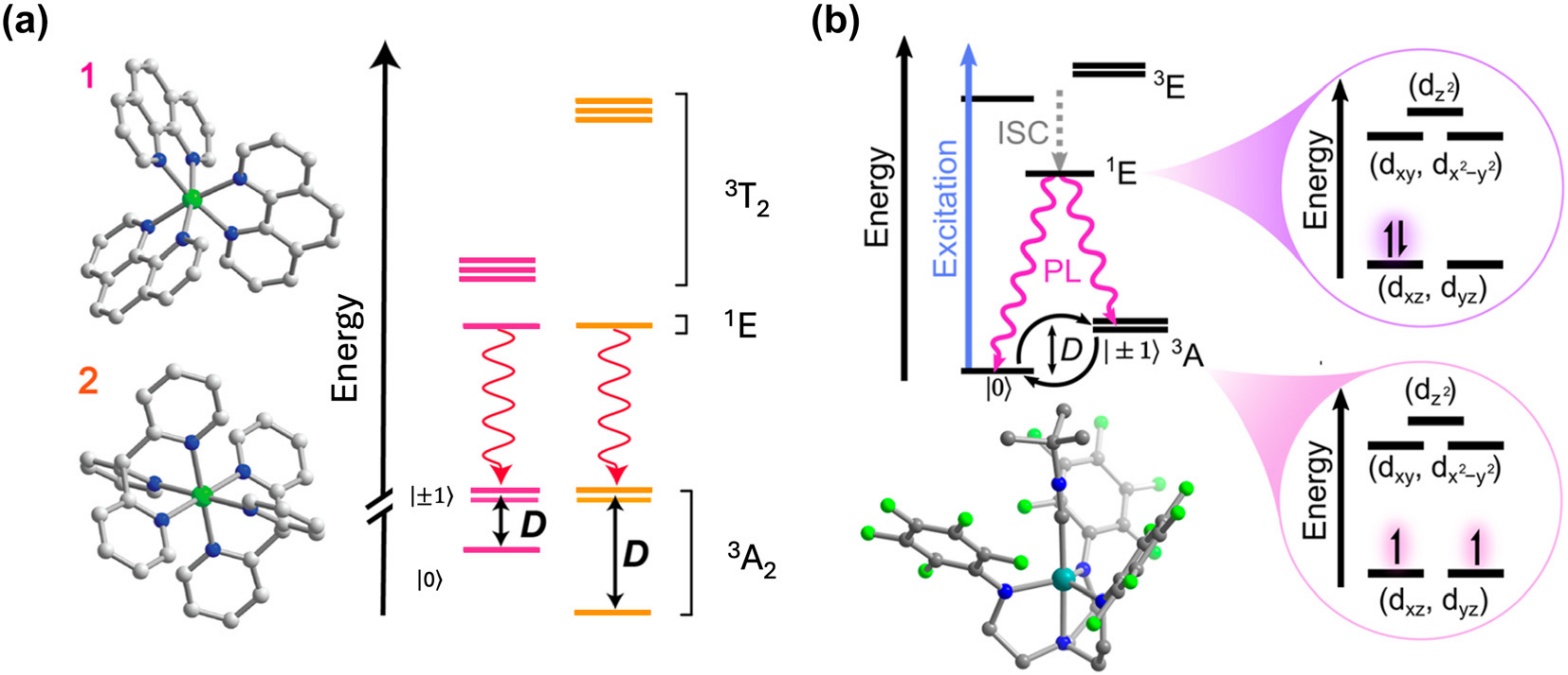
Ni^2+^ and V^3+^ complexes proposed as candidates for optically addressable molecular spin qubits. (a). Molecular structures and energy level diagrams of [Ni(phen)_3_](BF_4_)_2_] (1) and [Ni(pyr_3_)_2_](BF_4_)_2_] (2). Green, blue, and gray spheres represent Ni, N, and C atoms, respectively. H atoms are omitted for clarity. The figure is adapted with permission from ref. [67]. (b) Illustration of the energy level scheme for [(C_6_F_5_)_3_trenVCN^t^Bu], highlighting the spin conserving optical excitation (blue), intersystem crossing (ISC) to the ^1^E (*S* = 0) excited state (gray), and photoluminescence (PL) from the later to the ^3^A (*S* = 1) ground state. The molecular structure determined by single-crystal X-ray diffraction is shown below with teal, blue, green, and gray spheres representing V, N, F, and C, atoms, respectively. H atoms are omitted for clarity. Pink circles: Qualitative d-orbital splitting diagram and electronic configurations for ^1^E and ^3^A in ideal C_3v_ symmetry. The figure is adapted with permission [66].

Optically addressable TM-based spin qubit candidates composed of Ni^2+^ [67], [68], V^3+^ [66] and V^4+^ [75], [74] have also been reported (Table 4). Ni^2+^ and V^3+^ systems feature spin triplet (*S* = 1) ground state while V^4+^ complexes present spin doublet (*S* = 1/2). The design of Ni^2+^ complexes must account for the large spin–orbit constant; therefore, a high sensitivity to ligand field often yields a large zero-field splitting. By leveraging a quasi-octahedral coordination symmetry, Wojnar and co-authors reported two Ni^2+^ complexes – [Ni(phen)_3_](BF_4_)_2_] and [Ni(pyr_3_)_2_](BF_4_)_2_] (with phen = 1,10-phenanthroline and pyr_3_ = tris-2-pyridyl-methane) – fulfilling the EPR addressability criteria, as well as optical emission occurring between 938 and 944 nm [68] (Figure 6(a)). As far as vanadium is concerned, it features two interesting attributes – intrinsic nuclear spin I = 7/2, potentially useful for long-term quantum storage [3], and infrared (IR) emission lines compatible with fiber optic networks [76]. The proposal in ref. [66] is a trigonal bipyramidal V^3+^ complex: [(C_6_F_5_)_3_trenVCN^t^Bu], where ‘tren’ stands for tris(2-aminoethyl)amine) (Figure 6(b)). High-frequency EPR addressability (240 GHz) was confirmed in this compound as well as a zero-phonon optical transition at 1,237 nm with a linewidth comparable to the spin zero-field splitting, allowing for a selective optical addressing of the spin transitions under high magnetic field (>6 T) [66]. Another family of vanadium-based complexes that have shown good prospects as electron spin qubits, due to their long *T*_2_^e^ times, are those based on V^4+^ [75]. Among them, the recently-reported vanadyl complex [VO(bdhb)] (with bdhb = 1,3-bis(3,5-dioxo-1-hexyl)benzene) [74] (Entry 3; Table 4), shows EPR addressability in the X-band (9.4 GHz), and is attractive because of its neutral charge, nuclear spin-free donors, and potential for chemical functionalization on the aromatic ring.

In conclusion, Ni^2+^, V^3+^ and V^4+^ complexes could emerge as attractive TM-based qubit platforms due to their added functionalities – for instance, optical addressability within the IR region, compatible with optical fiber networks. Yet, they are still behind the best Cr^4+^ complexes [21], [65] in terms of *T*_2_^e^ (Entries 2–5; Table 4) and optical spin manipulation capabilities.

Finally, TM complexes exhibiting biocompatibility and chemical stability are also developped for quantum sensing applications. For a molecular system to function as a quantum sensor, it should satisfy the same design criteria laid out for optically addressable molecular spin qubits, i.e. EPR addressability combined with optical initialization and readout capacities. Compounds proposed for quantum sensing include the Ni^2+^ complex [Ni(ttcn)_2_](BF_4_)_2,_ featuring a thioether-based ttcn ligand (ttcn = 1,4,7-trithiacyclononane) [68] (Entry 6; Table 4).

## 4 Rare-earth ion (REI) complexes as photonic quantum materials

The rare-earths/lanthanides are a group of 17 elements including the lanthanides, scandium, and yttrium. Trivalent lanthanides present [Xe]4f^n^ electronic configuration giving rise to many energy levels between which radiative transitions may occur. These emissive 4f levels are shielded by the outer 5s and 5p orbitals so that they are not strongly affected by the coordinating ligands. At low temperatures, REIs doped into highly crystalline host lattices can show very long optical coherence time, *T*_2_^opt^ [77], as well as exceptionally long electron (*T*_2_^e^) and nuclear (*T*_2_^*n*^) spin coherence times [13], [15], [78]. Consequently, REI-doped host materials have been used to realize quantum memories for light [79], [80] and light–matter teleportation [81]. They are also suitable for quantum computing applications [82], [83]. Embedding REIs into molecular hosts could yield REI materials featuring desirable qubit figures of merits that are tuneable following molecular engineering approaches. Such possibilities are the driving force behind the studies of REI-based molecules as photonic quantum materials.

### 4.1 Europium-based molecular crystals for coherent spin-photon interfaces

**Figure 7:**
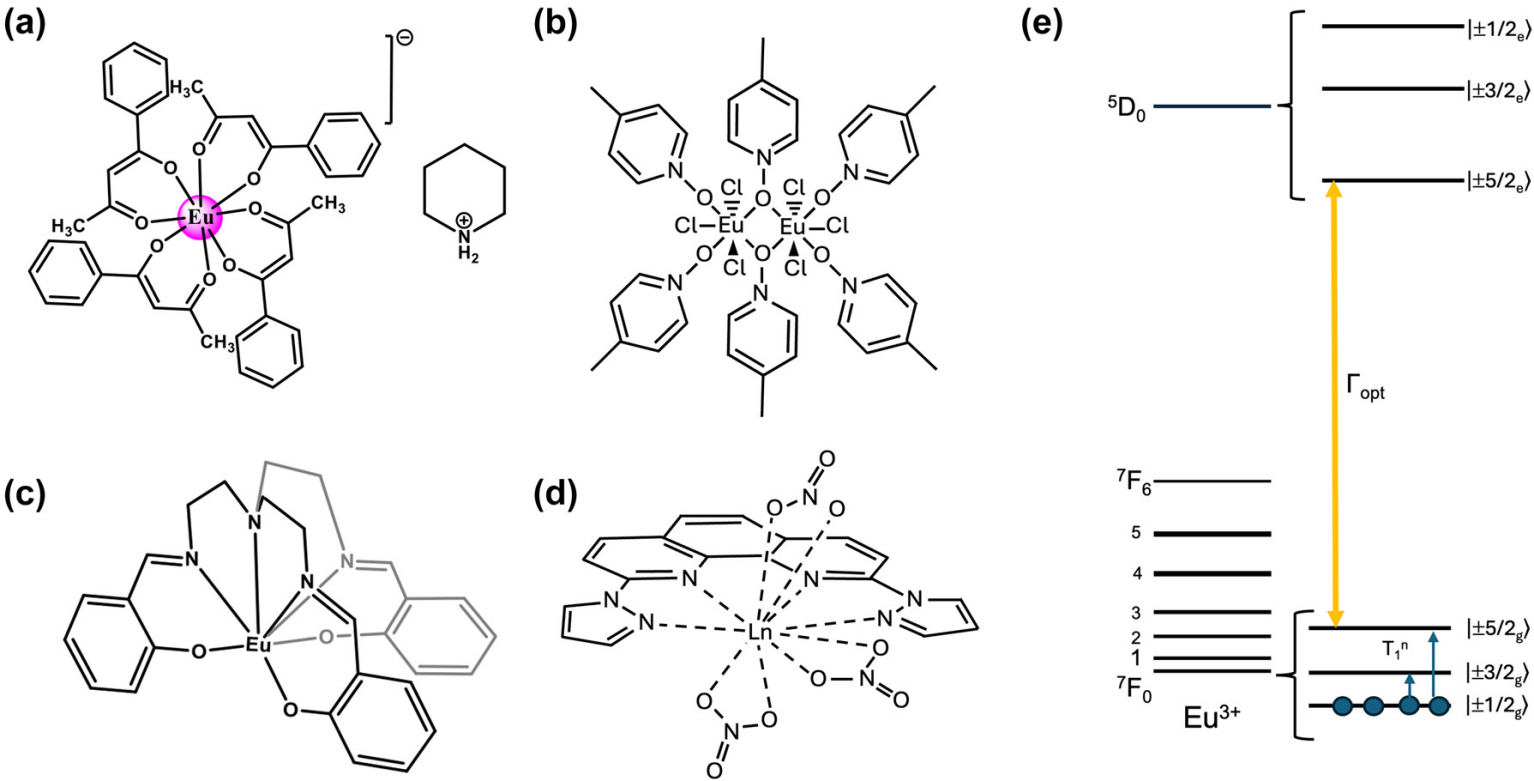
Europium (Eu^3+^) complexes show narrow optical homogeneous linewidths (Γ_h_^opt^) and long nuclear spin relaxation times (*T*_1_^*n*^): (a) eight coordinate mononuclear [Eu(BA)]_4_(pip) (BA = benzoylacetonate; pip = piperidin-1-ium), (b) seven coordinate binuclear [Eu_2_Cl_6_(4-picNO)_4_(*μ*_2_-4-picNO)_2_]·2H_2_O] (4-picNO stands for 4-picoline-N-oxide), (c) seven coordinate [Eu(trensal)] (trensal = 2,2′,2″-tris(salicylideneimino)triethylamine), and (d) ten coordinate [Eu(dpphen)(NO)_3_] (dpphen = 2,9-bis(pyrazol-1-yl)-1,10-phenanthroline), with O_8_, O_4_Cl_3_, O_3_N_4_, and O_6_N_4_ coordination environments, respectively. (e) Electronic levels of Eu^3+^ – the narrow optical transition occurs between the ^5^D_0_ and ^7^F_0_ levels – and nuclear spin structure of the two stable isotopes – ^151^Eu and ^153^Eu – of Eu^3+^ composed of three doubly degenerated levels in the ground and excited states. In the scheme, nuclear spin population polarization by optical pumping followed by spin relaxation causing population equilibrium is represented.

**Figure 8:**
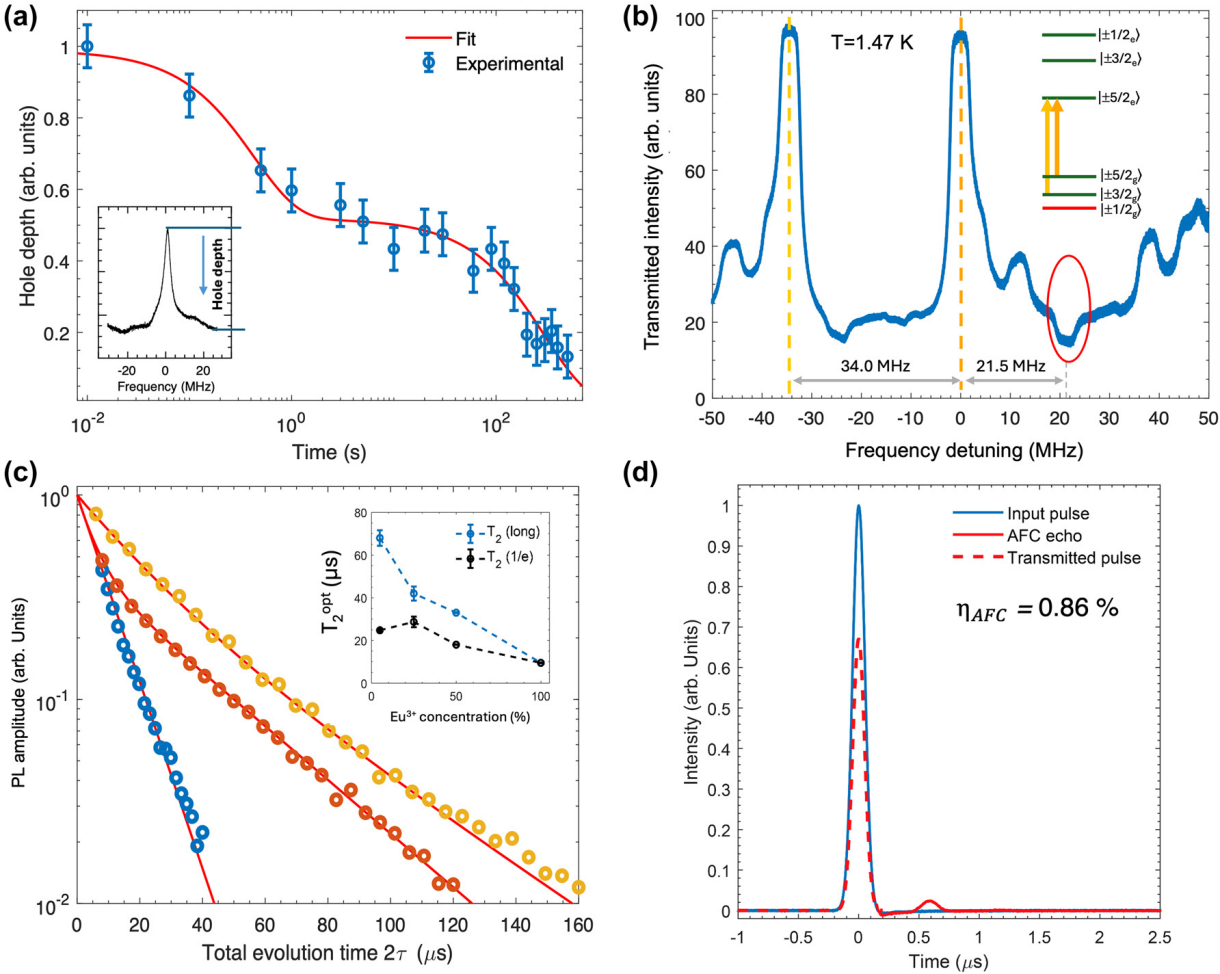
Time-dependent hole decay, optical polarisation of nuclear spin states, optical *T*_2_ extension by dilution and coherent storage in Eu(BA)]_4_(pip). (a) Nuclear spin relaxation time (*T*_1_^*n*^) estimated from the decay of the spectral hole depth as a function of time. Inset: detail of a spectral hole. The vertical axis corresponds to transmission. The arrow illustrates the decay of the hole depth with time due to the relaxation of the nuclear spin population. (b) Spin initialization to a single nuclear spin level in [Eu(BA)]_4_(pip) [25]. (c) *T*_2_^opt^ extension by dilution into [Y(BA)]_4_(pip), revealing dephasing due to electric dipole-dipole interactions between the Eu^3+^ centres in the fully concentrated [Eu(BA)]_4_(pip) molecular crystal. (d) Optical storage of a Gaussian pulse with 0.86 % efficiency using the atomic frequency comb (AFC) quantum memory protocol. Adapted with permission from ref. [25].

Narrow Γ_h_^opt^ – from few tens of MHz and down to 30 kHz – combined with long nuclear spin relaxation times (*T*_1_^*n*^) – up to several hundreds of seconds – have been reported for molecular europium (Eu^3+^) complexes (Figure 7 and Table 5) in their stoichiometric crystalline form [25], [84], [85], [86]. The transition of interest is the ^5^D_0_ → ^7^F_0_ transition centered about 580 nm. This transition is forbidden by selection rules and only takes place when the Eu^3+^ center is placed in a low-symmetry coordination environment [87], [88], establishing a first design criteria for Eu^3+^ complexes. Eu^3+^ presents two stable isotopes, ^151^ and ^153^, both with nuclear spin *I* = 5/2, giving rise to three doubly degenerated nuclear spin levels at zero magnetic field. The optical and spin transitions of REI are in general less sensitive to their environment than those of transition metal complexes. Yet, as displayed in Table 5, the optical coherence properties of Eu^3+^ complexes can significantly vary from one molecule to another. For instance, at 1.4 K, a Γ_h_^opt^ of 22 MHz (i.e. 14.5 ns *T*_2_^opt^) was observed for a seven coordinate binuclear Eu^3+^ complex (Figure 7(b) and Entry 4; Table 5) [84], while a Γ_h_^opt^ of only 30 kHz (10.5 μs *T*_2_^opt^) was observed for the eight coordinate mononuclear Eu^3+^ complex [Eu(BA)]_4_(pip) (BA = benzoylacetonate; pip = piperidin-1-ium) (Figure 7(a) and Entry 1; Table 5), a value several orders of magnitude narrower than the ones observed for other molecular emitters [18], [25], [69]. For the so far studied Eu^3+^ molecules, the obtained Γ_h_^opt^ is narrower than the ground-state nuclear spin splittings, allowing for optical addressing of the nuclear spin states of ion ensembles within the optical inhomogeneous line Γ_inh_^opt^. This was harnessed to probe the nuclear spin relaxation times *T*_1_^*n*^, which are in the seconds to hundreds of seconds regime (Table 5 and Figure 8(a)). It is interesting to note that the *T*_1_^*n*^ values are not directly correlated to the optical Γ_h_^opt^ values, as observed for the longer *T*_1_^*n*^ but broader Γ_h_^opt^ obtained for the Eu(trensal) complex (Entry 2; Table 5). This indicates different limiting underlying mechanisms contributing to optical line broadening and spin relaxation.

**Table 5:** Compilation of spectroscopic properties of Eu^3+^ complexes investigated as optically addressable molecular qubit candidates. Given values are at 1.4 K for [Eu(BA)]_4_(pip) and [Eu_2_], while at 4.2 K for Eu(trensal) and Eu(dpphen)(NO)_3_. Γ_inh_^opt^ stands for optical inhomogeneous linewidth, *T*_1_^opt^ for excited state population lifetime, Γ_h_ for optical homogeneous linewidth, *T*_2_^opt^ for optical coherence time, with Γ_h_^opt^ = (*πT*_2_^opt^), and *T*_1_^*n*^ for spin population time). These studies were all carried out in fully concentrated Eu^3+^ molecular crystals.

Entry	Molecule	Site	Γ_inh_^opt^	*T*_1_^opt^	Γ_h_^opt^	*T*_2_^opt^	*T*_1_^*n*^ (s)^c^	Ref.
1	[Eu(BA)]_4_(pip) **Figure:** 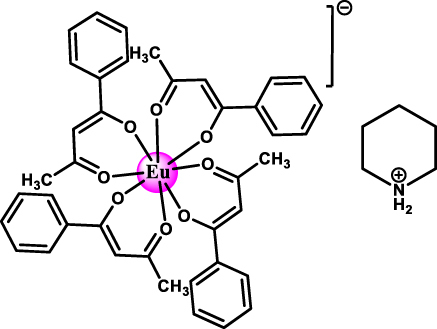	C_2v_	6.6 GHz	0.54 ms	30 kHz^a^	10.5 μs^a^	0.43/300	[25]
2	Eu_2_Cl_6_(4-picNO)_4_(*μ*_2_-4-picNO)_2_]·2H_2_O **Figure:** 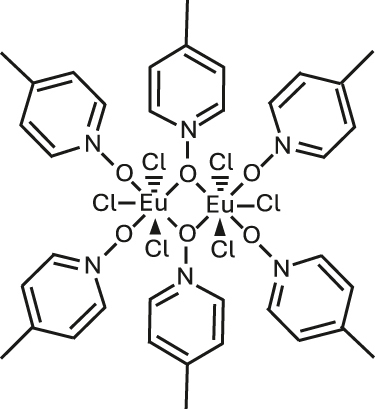	D_5h_^b^	50 GHz	0.88 ms	22 MHz	14.5 ns	1.6/>30	[84]
3	Eu(trensal) **Figure:** 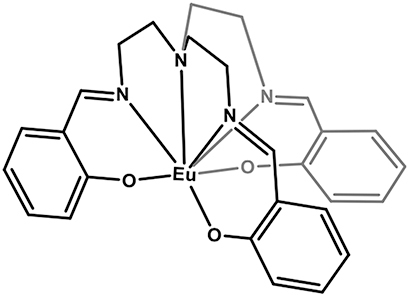	C_3v_	91 GHz	0.35 ms	2.8 MHz	114 ns	7/480	[85]
4	Eu(dpphen)(NO)_3_ **Figure:** 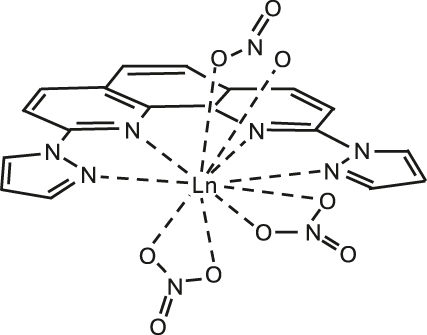	C_2v_	16 GHz	1.4 ms	3.1 MHz	102 ns	0.3/41	[86]

^a^From photon echo measurements. The rest of values were estimated from spectral hole widths. ^b^Site symmetry for Eu^3+^ in Eu_2_Cl_6_(4-picNO)_4_(*μ*_2_-4-picNO)_2_]·2H_2_O identified as distorted pentagonal bipyramidal. See ref. [84] for more details. ^c^The two values correspond to the two distinct time constants observed in the spectral hole decay.

The very narrow homogeneous linewidth Γ_h_^opt^ and long *T*_1_^*n*^ observed for crystals composed of a mononuclear Eu^3+^ complex [Eu(BA)_4_](pip), allowed for further demonstrations of >95 % spin polarization into a single level (Figure 8(b)), coherent optical storage up to 1 µs using the atomic frequency comb (AFC) quantum memory protocol (Figure 8(c)), and controlled ion–ion interactions of use for high-bandwidth two-qubit quantum gates. Impact of the host on *T*_2_^opt^ was also evidenced by diluting [Eu(BA)_4_](pip) in [Y(BA)_4_](pip), leading to an increase in *T*_2_^opt^ by a factor 2–6, depending on the dilution ratio, confirming optical dephasing due to electric dipole interaction between Eu^3+^ ions (Figure 8(d)).

### 4.2 Enhanced optical addressing and detection of REI molecules

The low sensitivity of REI to environmental perturbations is of use for achieving record-long optical coherence times in REI materials at low temperatures, a feature that distinguishes these materials from other optically addressable spin platforms. It comes however with the drawback of low emission rates for REI-based optical transitions making the readout of single REI difficult. Some degree of Purcell enhancement is therefore a requirement for optically addressing and detecting single REIs in an efficient manner for QIP applications [12], [17], [80], [89]. In the following we provide two examples showing that the natural compatibility of molecules with different photonic devices can be harnessed to obtain enhanced optical addressing and emission rates in REI complexes [90], [91].

**Figure 9:**
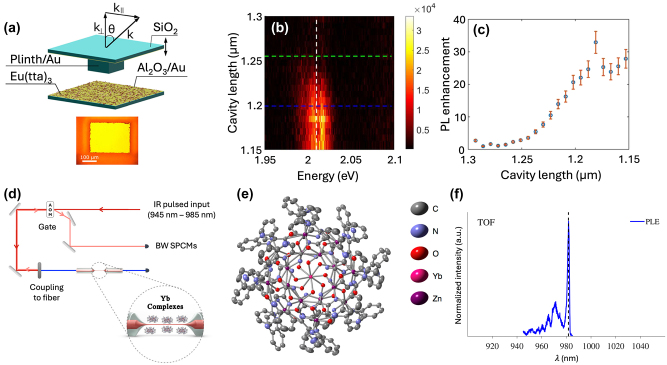
Photonic enhancement of optical addressing and detection of REI molecules. (a) Tunable open microcavity. The photonic structure is formed by two glass mirrors facing each other in the vertical direction and covered by 50 nm of Au and 5 nm of Al_2_O_3_. A layer of [Eu(TTA)_3_(DPEPO)] is deposited onto the bottom mirror of the cavity. Below – optical microscope image of the 200 μm × 300 μm plinth. (b) Cavity length-dependent PL spectra of the Eu^3+^ complex–cavity system at *k*_||_ = 0. The vertical dashed white line corresponds to the most prominent ^5^D_0_ → ^7^F_2_ transition. (c) PL enhancement factors derived from the white line in (b). Adapted with permission from ref. [90]. (d) Optical setup used to excite a monolayer of Yb^3+^ complexes coated on a tapered single-mode optical fiber. (e) Structure of the complex [Yb^3+^[Zn(II)_MC_(QXA)]], where MC and QXA stands for metallo crown and 2-Quinoxaline carboxylic acid, respectively, obtained by XRD (thermal ellipsoid structure presentation with a probability of 50 %; hydrogens are omitted for clarity). (f) PLE spectrum obtained from the TOF coated with a monolayer of the complexes at *T* < 10 K with a characteristic narrow absorption line around 980 nm attributed to Yb^3+^.

In the first example, enhanced photoluminescence (PL) has been reported by Emmanuele and co-authors [90] for the ^5^D_0_ → ^7^F_2_ transition of Eu^3+^ in the complex [Eu(TTA)_3_(DPEPO)], where DPEPO stands for bis(2-(diphenylphosphino)phenyl) ether oxide, via a Fabry–Pérot micro-cavity tuned in resonance with the transition. The Eu^3+^ complexes are deposited onto the bottom mirror of the cavity (Figure 9(a)). A maximum enhancement value of 30 is achieved for a cavity length of 1.2 μm (Figure 9(c) and (d)). From the shortening of the PL decay under cavity coupling, it is also concluded that the spontaneous emission rate is increased by a factor of 140. This emission acceleration is attributed to amplified spontaneous emission from the super-linear dependence of the PL intensity with the excitation power.

The second example, by Mor and co-authors [91] concerns the evanescent-field coupling of a monolayer of a highly radiative Yb^3+^ complex – [Yb^3+^[Zn(II)_MC_ (QXA)]], with lifetime >0.95 ms, coated on a tapered single-mode optical fiber (TOF) of ∼500 nm in diameter (Figure 9(d) and (e)). For this, the TOF’s surface was successfully functionalized to allow for the binding of the Yb^3+^ complexes (see ref. [91] for details). Optical addressing of the molecular monolayer was confirmed by monitoring the PL intensity coupled to the fiber as a function of the excitation wavelength, revealing the characteristic absorption maximum of Yb^3+^ ions around 980 nm (Figure 9(f)).

## 5 Discussion and perspectives

In this review we have presented a discussion on several classes of molecular platforms, and representative examples for each class, currently pursued for optically addressable spin qubits and spin-photon interfaces. This is an emerging research field, however landmark results have been obtained, setting the potential of these systems and pointing at the possible directions for future developments. In all discussed platforms, the ability of chemistry to tune the molecular structure and/or the environment in a rationale and reproducible manner is harnessed to improve the qubit system properties. This strong chemical tunability is particularly prevalent in the case of all-organic molecules. In this systems, the choice of the parts constituting the molecule is fundamental for creating spin multiplets that can be used as qubits (or qu*d*its), and that can be optically read out [33]. The photogenerated electronic spin states are often polarized by applying magnetic field and typically present electron spin *T*_2_^e^ values of several microseconds at relatively high temperatures (85 K) [56]. Although still far from the best electron spin *T*_2_^e^ values measured in other solid-state systems, they are sufficient to demonstrate two-qubit gate operations [55], and to proof the ability of some all-organic chromophore systems to be used as short-range interconnects [22]. Additionally, all-organic chromophores benefit from strong light absorption by the aromatic fragments. However, the absorption mostly occurs in the UV and visible ranges, making these molecular systems less adapted to directly convert photonic qubits (typically in the telecom range) into electron or nuclear spin qubits.

Transition-metal-ion complexes also benefit from strong chemical tunability to meet the desired qubit properties. This is demonstrated by the ability to judiciously tuning the TM electronic energy level structure and zero-field splitting by means of the ligand field and site symmetry [19]. All-optical polarization and readout have been demonstrated in designed Cr^4+^ complexes, as well as Rabi oscillations and electron spin *T*_2_^e^ values that can reach up to 10 μs at 4 K by leveraging host-design capabilities [21]. Besides, V^3+^ complexes offering optical addressing and readout in the NIR range, combined with relatively narrow optical linewidths have also been reported [66]. Finally, the so far studied REI complexes stand out by their exceptionally narrow optical homogeneous linewidths, down to 30 kHz (10.5 μs *T*_2_^opt^), and hundreds of seconds long nuclear spin relaxation times below 10 K, making them ideal candidates for quantum photon-spin interfaces [25]. This is due to the relatively low impact of the surrounding environment on the REI properties, which in turn makes these systems the less tunable among all discussed complexes. Yet, as demonstrated by the clearly distinct optical coherence performances of different Eu^3+^ complexes, the molecular structure and host design still plays a fundamental role toward obtaining the desired properties [84], [85]. The interest of the molecular approach in the case of REI has also been highlighted by the possibility to use short and well-defined qubit–qubit interaction distances [84], as well as enhanced light molecule-interactions by coupling to optical devices [91]. Demonstrations of coherent nuclear spin manipulations are still to be reported in Eu^3+^ complexes, as well as optical, electronic and nuclear spin coherence values in other REI-containing complexes – for example, Yb^3+^ and Er^3+^ complexes. The combination of NIR transitions, including at telecom wavelength, and electron and nuclear spin degrees of freedom make complexes containing V^3+/4+^, Yb^3+^, and Er^3+^ as ideal candidates for realizing photonic QIP architectures.

While the potential of all these molecular platforms for developing custom designed, reproducible, and scalable quantum hardware has been elucidated, important progress and developments are still required for these systems to meet the figures of merit achieved by more advanced QIP platforms, such as the already commercial superconducting qubits, or color centers in diamond [3], [9]. We are however confident that the required progress can be obtained, taking advantage of the large number of possible molecular structures, and the increasing understanding of the key design parameters toward achieving the desired quantum properties. In conclusion, the molecular road ahead opens a very exciting direction, in our opinion, worth embarking to develop qubit hosting platforms for QIP applications.
